# Role for carbohydrate response element-binding protein (ChREBP) in high glucose-mediated repression of long noncoding RNA Tug1

**DOI:** 10.1074/jbc.RA120.013228

**Published:** 2020-05-28

**Authors:** Jianyin Long, Daniel L. Galvan, Koki Mise, Yashpal S. Kanwar, Li Li, Naravat Poungavrin, Paul A. Overbeek, Benny H. Chang, Farhad R. Danesh

**Affiliations:** 1 Section of Nephrology, Division of Internal Medicine, The University of Texas at MD Anderson Cancer Center, Houston, Texas, USA; 2 Department of Nephrology, Rheumatology, Endocrinology and Metabolism, Okayama University Graduate School of Medicine, Dentistry and Pharmaceutical Sciences, Okayama, Japan; 3 Department of Pathology, Feinberg School of Medicine, Northwestern University, Chicago, Illinois, USA; 4 Department of Nephrology, The Second Xiangya Hospital, Central South University, Changsha, Hunan, China; 5 Department of Clinical Pathology, Faculty of Medicine Siriraj Hospital, Mahidol University, Bangkok, Thailand; 6 Department of Molecular and Cellular Biology, Baylor College of Medicine, Houston, Texas, USA; 7 Department of Pharmacology & Chemical Biology, Baylor College of Medicine, Houston, Texas, USA

**Keywords:** long noncoding RNA (lncRNA), taurine upregulated gene 1 (Tug1), carbohydrate response element-binding protein (ChREBP), transcription regulation, engineered soybean ascorbate peroxidase (APEX2), diabetic nephropathy, kidney, gene regulation, epigenetics, transcription, CRISPR/Cas, APEX2, ChREBP, Tug1

## Abstract

Long noncoding RNAs (lncRNAs) have been shown to play key roles in a variety of biological activities of the cell. However, less is known about how lncRNAs respond to environmental cues and what transcriptional mechanisms regulate their expression. Studies from our laboratory have shown that the lncRNA Tug1 (taurine upregulated gene 1) is crucial for the progression of diabetic kidney disease, a major microvascular complication of diabetes. Using a combination of proximity labeling with the engineered soybean ascorbate peroxidase (APEX2), ChIP-qPCR, biotin-labeled oligonucleotide pulldown, and classical promoter luciferase assays in kidney podocytes, we extend our initial observations in the current study and now provide a detailed analysis on a how high-glucose milieu downregulates Tug1 expression in podocytes. Our results revealed an essential role for the transcription factor carbohydrate response element binding protein (ChREBP) in controlling Tug1 transcription in the podocytes in response to increased glucose levels. Along with ChREBP, other coregulators, including MAX dimerization protein (MLX), MAX dimerization protein 1 (MXD1), and histone deacetylase 1 (HDAC1), were enriched at the *Tug1* promoter under high-glucose conditions. These observations provide the first characterization of the mouse Tug1 promoter's response to the high-glucose milieu. Our findings illustrate a molecular mechanism by which ChREBP can coordinate glucose homeostasis with the expression of the lncRNA Tug1 and further our understanding of dynamic transcriptional regulation of lncRNAs in a disease state.

Long noncoding RNAs (lncRNAs) are classically known as diverse RNA transcripts that are more than 200 nucleotides long and are not translated into proteins or encode very short peptides (1, 2). Recent advances in high-throughput DNA sequencing and single-cell RNA-Seq studies have revealed that lncRNAs have crucial roles in regulating gene expression and play broad roles impacting human physiology and pathophysiology (1, 3), yet despite the striking prevalence of lncRNAs and countless attempts to explore their function, the functional landscape of the majority of lncRNAs remains elusive (3–5).

Growing evidence suggests that lncRNAs play key roles in linking the metabolic state of the cell to extracellular cues and nutrient availability. For instance, we have recently shown that the lncRNA Tug1 is downregulated in the podocytes of diabetic mice and plays an important role in progression of diabetic nephropathy (6). Mechanistically, we found that Tug1 regulates the expression of PGC-1α (peroxisome proliferator-activated receptor gamma coactivator 1α), a master transcription regulator of mitochondrial biogenesis, under high-glucose (HG) stress conditions by binding to an enhancer region that is 400 kb upstream of the PGC-1α gene, serving as a bridge that connects the *cis*-element and *trans*-factor for the expression of this important mitochondrial master regulator. However, despite much progress in identifying the biological scope and the function of Tug1, our current understanding of the precise regulation of Tug1 and its upstream transcriptional regulatory mechanisms remains very limited.

lncRNAs are often expressed in tissue- and/or development-specific patterns (7, 8), suggesting that their expression is tightly regulated by a number of well-characterized transcription factors (7–9). In this study, we provide strong evidence indicating that the HG-mediated suppression of Tug1 expression is, at least in part, regulated by ChREBP (carbohydrate response element binding protein, also known as MLXIPL, for Mlx-interacting protein-like), a major glucose-responsive transcription factor (10).

Enriched in adipose tissue, ChREBP is a basic helix-loop-helix leucine zipper transcription factor that regulates glucose homeostasis and the response to dietary carbohydrates (11–13). Indeed, an HG environment promotes ChREBP translocation to the nucleus, leading to the formation of a heterodimeric complex with MLX (Max-like protein X) and binding to the carbohydrate response elements (ChoRE) of ChREBP target genes in the nucleus (14–16).

In the current study, we show that in response to HG milieu, ChREBP can recruit other members of the ChREBP network, including MLX, MAX (Myc-associated factor), MXD1 (Max dimerization protein 1), and HDAC1 (histone deacetylase 1), to a ChoRE element and neighboring E-box (enhancer box) in the Tug1 promoter, suppressing transcriptional activity.

## Results

### Genomic organization of mouse lncRNA Tug1 and its promoter

**Figure 1.**
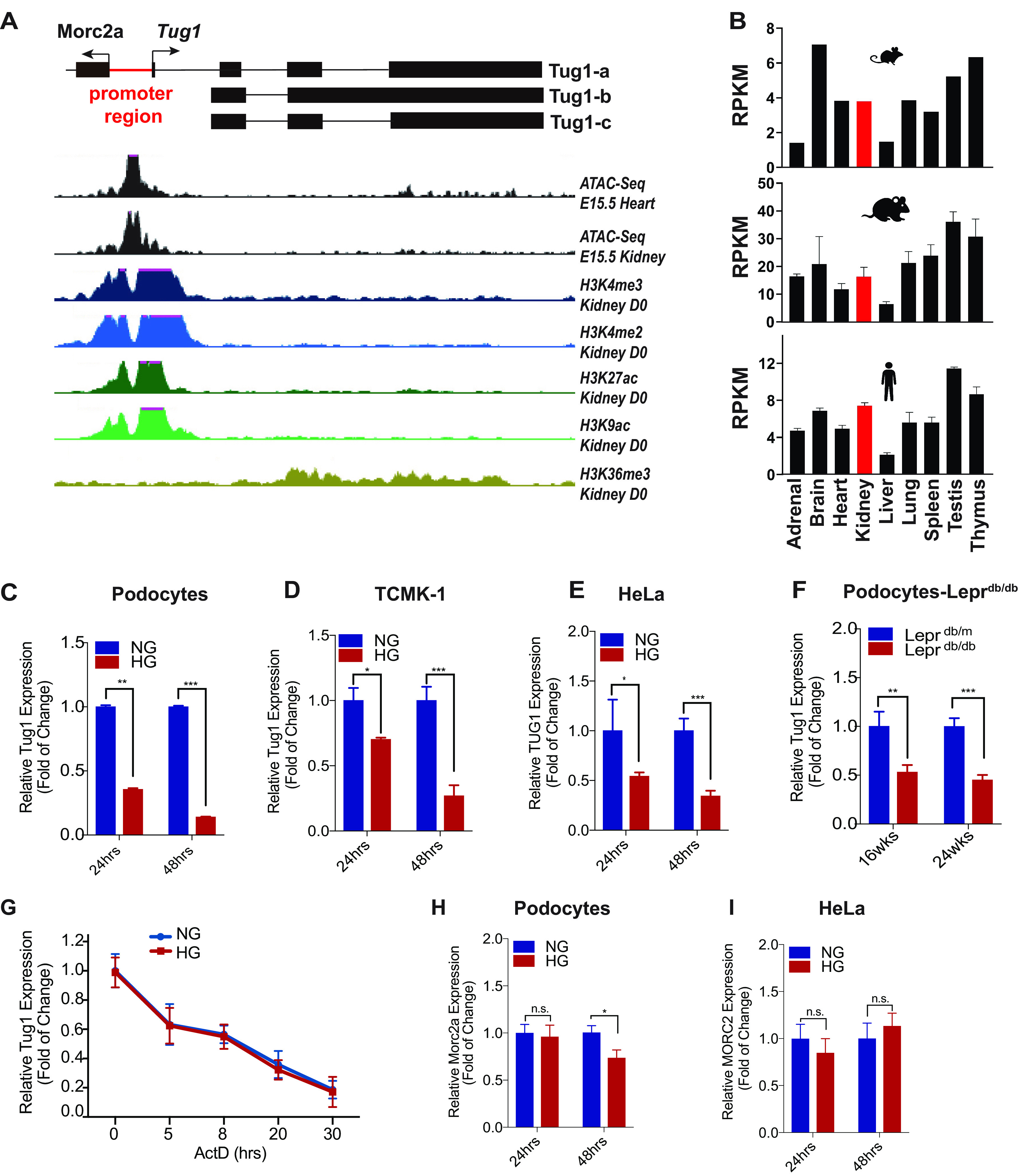
**Tug1 genomic locus, tissue expression pattern, and transcription regulation by high glucose.** *A*, selective UCSC Genome Browser tracks for ATAC-Seq (E15.5) and various histone 3 lysines methylation and acetylation patterns. *B*, Tug1 RNA expression in various tissues of mice, rats, and humans (human HPA RNA-Seq, rat RNA-Seq, and mouse ENCODE transcriptome data). *C–E*, RNA levels of Tug1 in mouse podocytes (*C*), transformed C3H mouse kidney-1 (TCMK-1) cell line (*D*), or HeLa cells (*E*) cultured under NG (5 mm) or HG (25 mm) conditions for the indicated time were analyzed by RT-qPCR (*n* = 3). Expression values were normalized to Gapdh. Data are presented as mean ± S.E.M. *, *p* < 0.05; **, *p* < 0.01; ***, *p* < 0.001. *F*, relative Tug1 RNA expression in primary podocytes isolated from leptin receptor-deficient heterozygous (Leprdb/m) and homozygous (Leprdb/db) mice at 16 or 24 weeks of age (*n* = 5 animals). *G*, podocytes cultured under normal glucose (NG; 5 mm) or high-glucose (HG; 25 mm) conditions were treated with actinomycin D (ActD) (4 mm) for the indicated time, and Tug1 levels were measured by qRT-PCR and normalized to Gapdh (*n* = 3). *H–I*, relative mouse Morc2a or human MORC2 mRNA levels in podocytes (*H*) or HeLa cells (*I*) cultured in NG and HG for the indicated time were analyzed by RT-qPCR (*n* = 3). Expression values were normalized to Gapdh or GAPDH. Data are presented as mean ± S.E.M. n.s., not significant. *, *p* < 0.05.

The human lncRNA *TUG1* gene (NCBI reference sequence NR_110492 transcript variant 1) is located on chromosome 22q12.2 and has 8 variant transcripts, ranging from 5.2–7.6 kilobase in length, whereas the murine *Tug1* lncRNA locus is located on chromosome 11 and has three variant (a, b, and c) transcripts that are 4.1–6.7 kilobases long (Fig. 1*A*). The *Tug1* promoter has *in vitro* transcriptional activity in both orientations and acts as a bidirectional promoter driving the expression of the protein-coding gene *Morc2a* as well (4, 17, 18). Consistent with a classical feature of bidirectional promoters, where the transcription start sites (TSS) of the two genes are predictably separated by less than 1 kb, the *Morc2a* TSS is separated by only 374 bp from the *Tug1* TSS. Importantly, *Tug1* is highly conserved among species and shows a wide tissue expression pattern in mice, rats, and humans (Fig. 1*B*) (4, 19). Moreover, the *Tug1* locus is enriched with key features of active transcription, such as open chromatin regions harboring transcription factors and histone modifications, including H3 lysine 4-trimethylation (H3K4me3) and H3K36me3 (Fig. 1*A*), suggesting active transcription along the *Tug1* gene body.

### Reduced expression of lncRNA Tug1 in several experimental models of diabetes is transcriptionally regulated

Previously, we had identified *Tug1* as an lncRNA that is downregulated in the diabetic milieu using an unbiased RNA-sequencing (RNA-Seq) analysis of kidney glomeruli (6). Here, we have expanded on our previous observations, finding significantly reduced expression of *Tug1* in several kidney cell lines that were cultured under HG conditions, including podocytes (Fig. 1*C*), TCMK-1 tubular cells (Fig. 1*D*), and HeLa cells (Fig. 1*E*). Notably, all three isoforms of Tug1 transcripts were downregulated under HG condition in podocyte (Fig. S1). We also validated that podocytes isolated from kidneys of leptin receptor-deficient (*db/db*) mice, an established model of type 2 diabetes, exhibited significant downregulation of *Tug1* expression compared with podocytes obtained from control nondiabetic (*db*/*m*) mice (Fig. 1*F*). We next argued that the *Tug1* downregulation could be because of diminished transcriptional activity or enhanced RNA degradation under high-glucose conditions. Therefore, we monitored *Tug1* RNA abundance by quantitative RT-PCR in the presence of actinomycin D, which intercalates into DNA, forming a stable complex and thereby inhibiting new RNA transcription (Fig. 1*G*). We found that the rate of RNA degradation was not significantly different between cells cultured under HG or NG conditions. This result suggests that the rate of *Tug1* degradation was similar in podocytes whether cultured under HG or NG conditions and indicates that HG-mediated downregulation of Tug1 was mainly because of its transcriptional regulation.

Because previous results had suggested that the Tug1 promoter acts as a bidirectional promoter driving the expression of Morc2a, we also determined whether HG has the same effect on Morc2a expression. To this end, cultured podocytes were exposed to HG for 24 h or 48 h (Fig. 1*H*). Unlike its neighbor Tug1 gene, which is transcribed in an opposite strand, we found that HG only modestly inhibited the expression of Morc2a at 48 h but not at 24 h. On the other hand, HG treatment did not have a significant effect on MORC2 mRNA expression in HeLa cells (Fig. 1*I*). These results suggest that HG does not have a consistent effect on Morc2a expression.

### ChoRE motif is present at the promoter region of Tug1 gene

**Figure 2.**
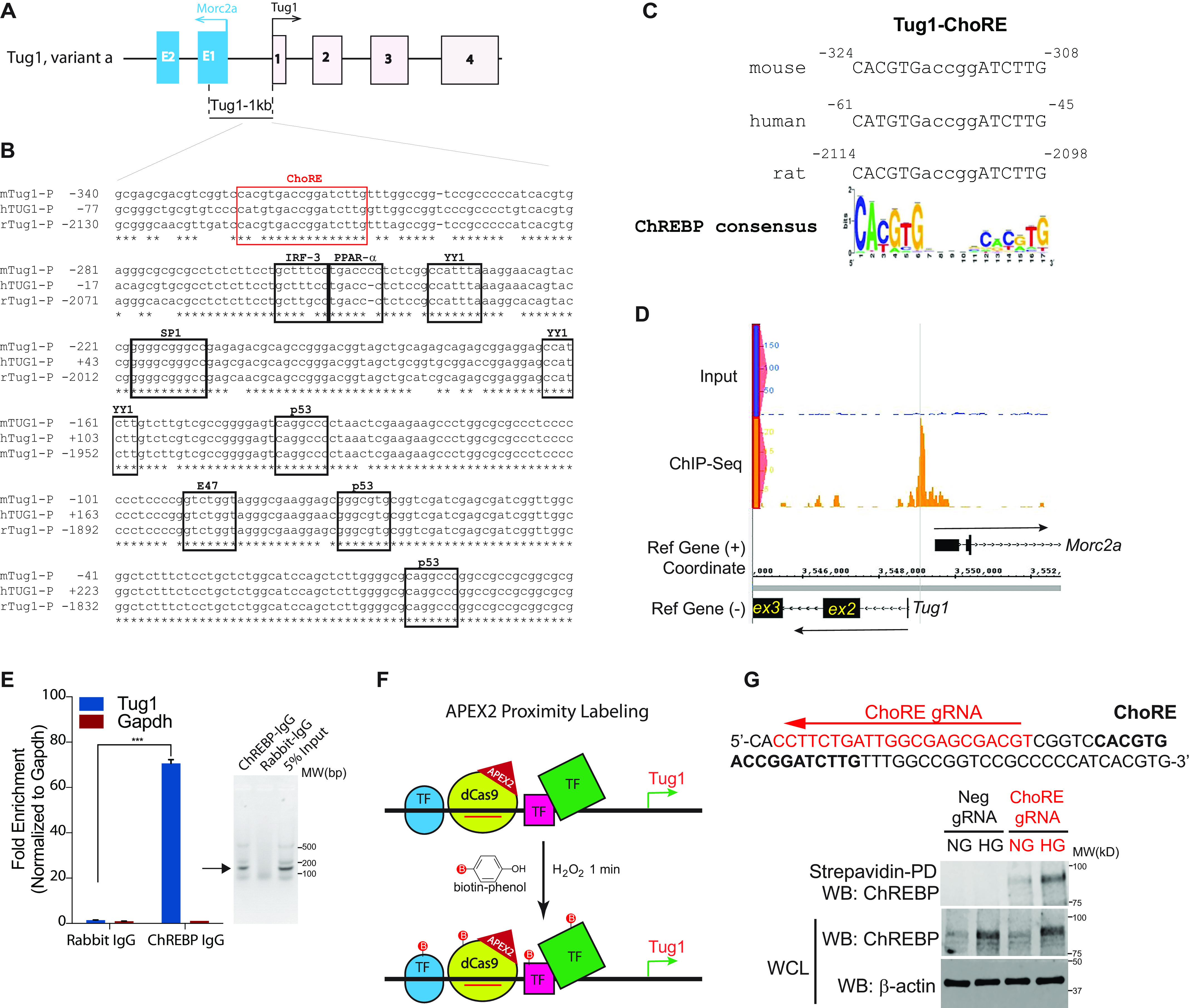
**Identification and validation of a consensus, conserved ChoRE in mouse Tug1 promoter region.** *A*, genomic structure of mouse Tug1 (variant a in GRCm38 assembly mm10) and the divergent gene Morc2a. 1 kb upstream of the Tug1 TSS is indicated. *B*, *in silica* analysis of 360 bp from the Tug1 proximal promoters in different species. Sequence alignment of Tug1 promoter regions from mouse (*m*), human (*h*), and rat (*r*). Consensus binding sites for the indicated transcription factors are shown. The potential ChoRE for ChREBP binding is highlighted in a *red box*. *C*, potential ChoREs in Tug1 promoters of mouse, human, and rat are consensus binding sites for ChREBP. *D*, ChREBP ChIP-Seq in mouse white adipocytes identified a specific peak centered at the potential ChoRE in the Tug1 promoter. *E*, *left*, ChIP-qPCR validation of ChREBP binding to Tug1 promoter in cultured mouse podocytes (*n* = 3). Data are presented as mean ± S.E.M. ***, *p* < 0.001. *Right*, analysis of ChIP-qPCR products on 1.2% agarose gel in sodium borate buffer. Expected bands are indicated with an arrow. *F*, a simplified diagram showing APEX2 peroxidase proximity labeling with a gRNA (*red line*) that guides a dCas9-APEX2 fusion protein to bind to the Tug1 promoter and mediates the oxidization of biotin-phenol in the presence of H_2_O_2_. Labeled proteins and transcription factors (*TF*; represented by *green*, *blue*, and *pink shapes*) are present in the proximity of Tug1 promoter-gRNA-dCas9-APEX2 complex. *G*, a gRNA that targets sequences adjacent to the ChoRE element in the Tug1 promoter results in ChREBP proximity labeling under high glucose. *Upper*, sequence of Tug1 promoter region with ChoRE element highlighted in *boldface* and the gRNA targeting site on the antisense strand highlighted in *red*. *Lower*, immunoblots of strepavidin pulldown (*PD*) or WCL from proximity labeling of stable cells cultured under NG or HG with a gRNA-targeting ChoRE (ChoRE gRNA) or a nontargeting gRNA (Neg gRNA).

To further explore how HG regulates *Tug1* transcription, we retrieved the promoter region of the murine *Tug1* gene to identify potential binding sites for transcription factors. Alignment of mouse, human, and rat *Tug1* promoter sequences indicated that these sequences are highly conserved, with over 90% of the sequences identical among these three species (Figs. 2*A*–*C*). Using a web-based tool, rVista 2.0, we identified several potential binding sites for transcription factors (Fig. 2*B*). Notably, we found multiple p53 candidate binding sites as well as SP1, YY1, E47, IRF-3, and PPAR-α binding sites. Importantly, we also found one ChoRE consensus sequence in this region that was almost identical in the three species we analyzed (Fig. 2*B* and *C*). The ChoRE sequence matched well with the consensus motif where two E-boxes (CAYGYG and CRCRTG) are separated by 5 nucleotides (where Y denotes pyrimidine [C or T] and R denotes purine [A or G]) (10, 20–22). Identifying ChoRE as a potential signature motif in the region was of great interest to us, because previous reports had demonstrated that ChoRE is recognized by the transcriptional factor ChREBP, a key transcription factor that is upregulated under HG conditions (10, 11).

We then screened whether the Tug1 promoter serves as a ChREBP binding site by taking advantage of genome-wide ChIP-Seq (ChIP coupled with high-throughput DNA sequencing) data from one of our previous studies of adipose tissue (21). Using model-based analysis of ChIP-Seq (MACS) (23), we detected a peak of ChREBP binding site at 324 bp upstream of the Tug1 promoter (Fig. 2*D*).

To mechanistically validate binding of ChREBP to the promoter of Tug1 under HG conditions, we used a ChIP assay by incubating nuclear extracts of cultured podocytes grown under HG with a rabbit antibody against ChREBP. As shown in Fig. 2*E*, only ChREBP-IgG, but not the control rabbit IgG, enriched Tug1 promoter chromatin DNA, which was detected by PCR amplification, suggesting that ChREBP indeed binds to the Tug1 promoter under HG conditions.

To further validate binding of ChREBP to the Tug1 promoter in live cells and to identify the regulatory factors that could potentially form a complex with ChREBP to control Tug1 expression under HG conditions, we used a combination of CRISPR targeting and proximity labeling with an engineered ascorbate peroxidase (APEX2) technology (24–26). We engineered a *piggyBac* vector to express U6 RNA promoter-driven guide RNA (gRNA) that targets the Tug1 promoter region and a dead Cas9 (dCas9)-APEX2 fusion construct under a CMV enhancer/promoter (27) (see Fig. S2 for construct design and sequence). We transfected this construct together with *piggyBac* transposase (28, 29) into the cultured podocytes. As depicted in Fig. 2*F*, the gRNA directs the fusion protein dCas9-APEX2 to the Tug1 promoter region. In the presence of H_2_O_2_, APEX2 will oxidize biotin-phenol into biotin-phenoxyl radicals (27, 30). The biotin-phenoxyl radicals can tag and attach to nearby proteins (∼20-nm radius) (31). We used magnetic conjugated streptavidin beads to enrich the biotinylated proteins in cultured podocytes. As a control, we used podocytes that expressed dCas9-APEX2 with a nontargeting gRNA. We proceeded to assess the biotinylated proteins labeled by dCas9-APEX2 in the gRNA-targeted Tug1 promoter region. Immunoblotting against ChREBP showed that ChREBP was biotinylated only in the cells transduced by the Tug1 promoter-targeted gRNA but not in the control podocytes (Fig. 2*G*). Importantly, we also confirmed that ChREBP is present at the Tug1 promoter and HG increases its prevalence (Fig. 2*G*).

### ChREBP suppresses Tug1 transcription through the ChoRE motif in Tug1 promoter

**Figure 3.**
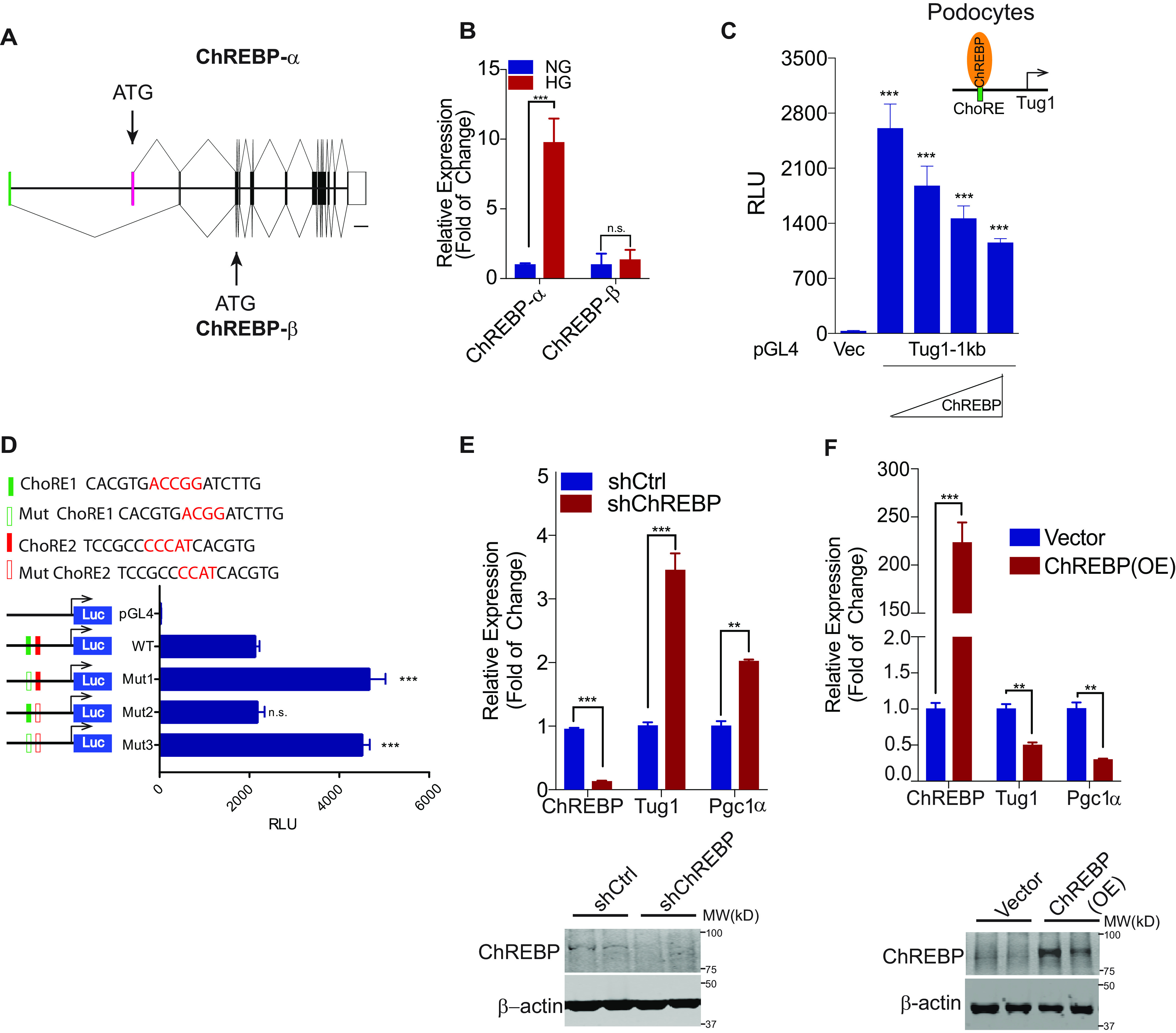
**ChREBP represses Tug1 transcription through the consensus ChoRE in the Tug1 promoter.** *A*, schematic depiction of two major murine ChREBP transcript variants, ChREBP-α and ChREBP-β. Each has its own transcription initiation (*green* and *pink* exons) and translation start sites (*arrows*). The graphs were drawn using the genomic DNA sequence through a web-based exon-intron graphic maker (http://wormweb.org/exonintron). *Scale bar*, 2,000 bp. *B*, relative ChREBP-α and ChREBP-β mRNA expression in culture podocytes grown under NG (5 mm) or HG (25 mm) conditions (*n* = 3). *C*, luciferase activities in podocytes cotransfected with Tug1-1kb promoter reporter construct (Tug1-1kb) or pGL4 vector (Vec) and increasing amounts of ChREBP-α cDNA expression constructs (*n* = 3). Luciferase readings are presented as mean ± S.E.M. ***, *p* < 0.001 (compared with vector group). *D*, ChoRE-1 (*green bar*) but not ChoRE-2 (*red bar*) is responsible for ChREBP-mediated repression of Tug1 transcription. *Top*, sequences of WT (*solid bars*) and ChoRE mutants (*blank bars*), where the 5-nt spacer between two E-boxes is mutated to 4 nt. *Bottom*, mouse podocytes were transfected with the indicated Tug1-1kb promoter constructs under normal glucose for 48 h, followed by luciferase assays (*n* = 3). Luciferase readings are presented as mean ± S.E.M. ***, *p* < 0.001 (compared with WT group); *n.s.*, not significant. *E–F*, Tug1-Pgc1α axis is regulated by loss-of-function and gain-of-function ChREBP. Tug1 and its downstream Pgc1α expression were analyzed by qRT-PCR in stable podocyte clones that were transfected with an shRNA against ChREBP (shChREBP) or a nontargeting control (shCtrl) (*E*) or PiggyBac-transposase-mediated integration overexpression of ChREBP [ChREBP(OE)] or vector control (Vector) (*F*) under normal glucose (*n* = 3). Expression values were normalized to Gapdh. Data are presented as mean ± S.E.M. **, *p* < 0.01; ***, *p* < 0.001. The efficiency of ChREBP knockdown and overexpression was demonstrated by immunoblots against ChREBP using β-actin as a loading control (*lower panels*). Results from two representative cell clones are shown in each group.

Because mouse ChREBP has two different transcript variants (61), ChREBP-α and ChREBP-β (Fig. 3*A*), we next examined which isoform is HG responsive in the podocyte cell line. Using quantitative RT-PCR and isoform-specific primers, we found that only the ChREBP-α transcript is upregulated under HG conditions (Fig. 3*B*). To explore the function of ChREBP on Tug1 transcription *in vitro*, we generated a luciferase expression construct driven by the 1-kb Tug1 promoter and cotransfected it with a construct expressing WT ChREBP-α. In the luciferase reporter assay, cultured cells transfected with a pGL4 control vector had negligible luciferase readings (Fig. 3*C*), but the vector containing the 1-kb Tug1 promoter gave significant luciferase activity. This promoter activity was suppressed by cotransfection with the ChREBP-α cDNA construct in a dose-dependent manner (Fig. 3*C*), suggesting that ChREBP can repress Tug1 transcription. Using luciferase reporter assays in podocytes, we also compared the effect of two ChREBP isoforms on Tug1 transcription and found that ChREBP-β is much less potent for repressing Tug1 transcription than ChREBP-α (Fig. S3*A*). Furthermore, we found transcription factor p53 significantly activates, whereas YY1 represses, Tug1 transcription (Fig. S3*B*), consistent with the presence of consensus binding sites for these transcription factors in the promoter region (Fig. 2*B*) and previous reports about the induction of Tug1 expression by p53 (32, 33). Because there are two potential ChoREs present in the promoter region of the murine Tug1 gene, we also generated several ChoRE mutant constructs where the 5-nucleotide (nt) spacer between the two direct repeat (DR1) motifs was shortened to 4 nt that should abolish the binding of ChREBP to either ChoRE1 or ChoRE2 (10, 34). The mutant ChoRE1 (mut1) failed to show suppression of the Tug1 promoter activity (Fig. 3*D*). The double mutant (mut3) in which single-nucleotide deletion occurred in both ChoRE1 and ChoRE2 showed promoter activity similar to that of the ChoRE1 mutant. In contrast, mutant ChoRE2 (mut2) showed the same level of promoter activity as the WT (Fig. 3*D*). These results indicate that ChoRE1, but not ChoRE2, plays an important role in ChREBP-mediated repression of Tug1 expression.

We further used loss-of-function and gain-of-function approaches to examine the role of ChREBP in the transcriptional regulation of the *Tug1* gene. We first knocked down ChREBP expression by shRNA in podocytes (Fig. 3*E*). Consistent with our previous observations, we found that ChREBP knockdown led to enhanced expression of Tug1 and its downstream target gene, Pgc1α. The knockdown efficiency was shown by Western blotting (Fig. 3*E*). Conversely, ChREBP overexpression led to the repression of both Tug1 and Pgc1α genes (Fig. 3*F*). These results suggest that lncRNA Tug1 is a target gene of transcription factor ChREBP.

### Identification of a repressor complex at the Tug1 promoter

**Figure 4.**
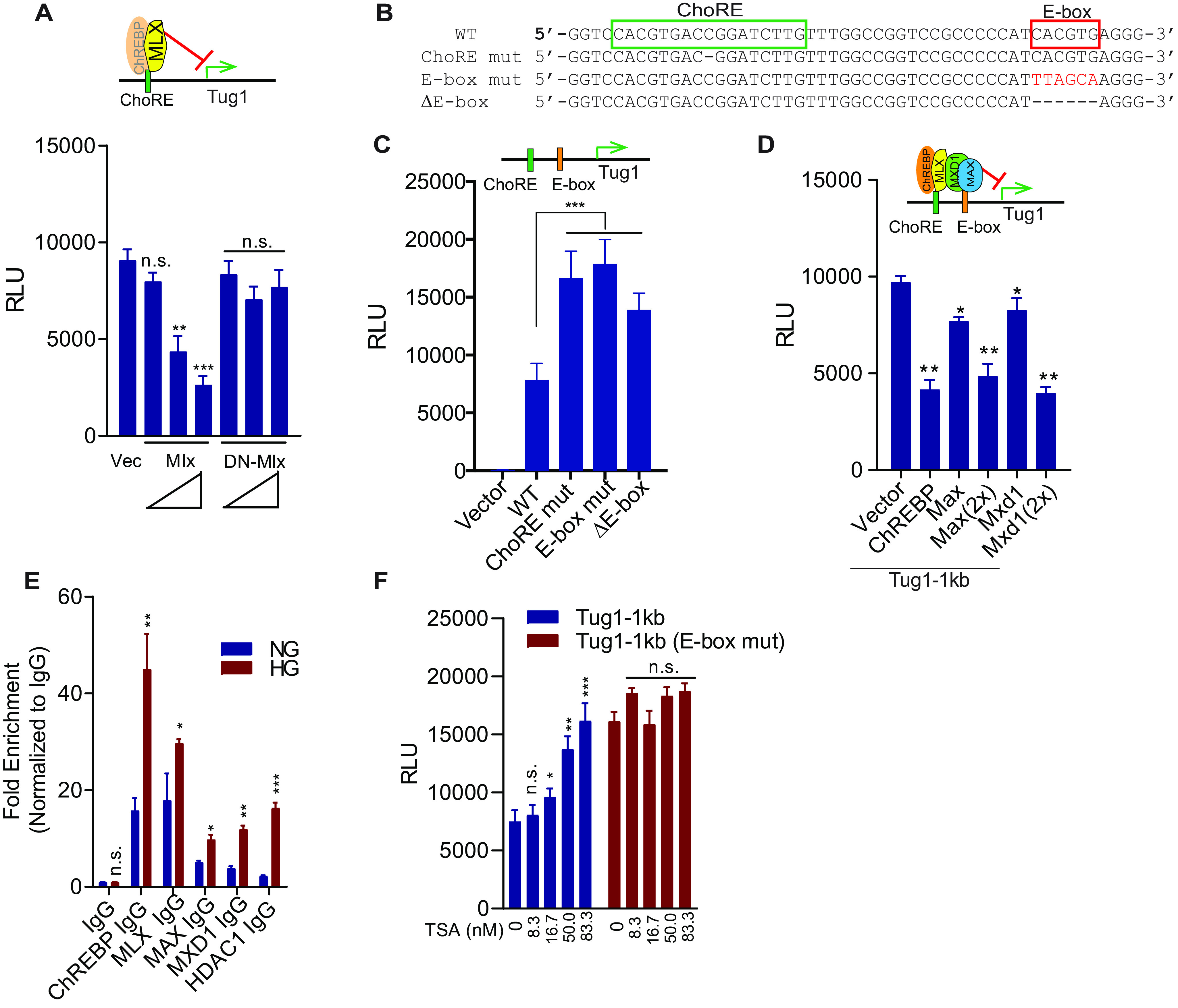
**MLX and MAX/MDX1 are recruited to Tug1 promoter to coordinate Tug1 repression.** *A*, MLX but not its dominant-negative mutant (DN-MLX) can repress the transcription of Tug1. Cultured mouse podocytes were transfected with Tug1-1kb promoter luciferase construct, together with the indicated Mlx overexpression plasmids or empty vector (Vec) under normal glucose conditions (*n* = 3). Luciferase readings are presented as mean ± S.E.M. **, *p* < 0.01; ***, *p* < 0.001 (compared with vector group); *n.s.*, not significant. *B*, sequence of glucose-responsive ChoRE and its neighboring E-box in the promoter of mouse Tug1 in WT, ChoRE mutant, E-box mutant, and E-box deletion mutant (ΔE-box). *C*, both ChoRE (*green bar*) and E-box (*orange bar*) bring repressive transcription factors to downregulate Tug1 transcription. Cultured mouse podocytes were transfected with the indicated luciferase constructs of Tug1-1kb promoter (WT), ChoRE and E-box mutants, or empty pGL4 vector (Vec) under normal glucose conditions. Luciferase assays were performed as in *A* (*n* = 3). *D*, MAX and MXD1 can repress Tug1 transcription. Cultured mouse podocytes were transfected with the Tug1-1kb promoter luciferase construct (Tug1-1kb), together with plasmids for the overexpression of the indicated transcription factors or empty pRK5 vector (Vector) under normal glucose conditions. Luciferase assays were performed as in *A* (*n* = 3). *E*, ChIP-qPCR analysis of Tug1 promoter region by the indicated antibodies in podocytes cultured under NG or HG conditions (*n* = 3). Data are presented as mean ± S.E.M. *, *p* < 0.05; **, *p* < 0.01; ***, *p* < 0.001; *n.s.*, not significant (compared with NG group). *F*, E-box is required for HDAC1-mediated transcription repression of Tug1. Cultured mouse podocytes were transfected with the indicated Tug1-1kb promoter luciferase constructs (WT or E-box mutant) and treated with the indicated concentrations of the HDAC inhibitor TSA or vehicle DMSO for 24 h under normal glucose conditions. Luciferase assays were performed as in *A* (*n* = 3).

ChREBP is known to bind to the ChoRE with another transcription factor, MLX, a ChREBP obligate partner, forming a heterodimer (14–16). Using the promoter-reporter system of the Tug1-1kb promoter luciferase construct in podocytes, we observed that Mlx dose-dependently suppressed the Tug1 promoter activity. A dominant-negative Mlx, however, did not exert a suppressive effect on the Tug1 promoter (Fig. 4*A*). We also identified a consensus E-box (enhancer box) (5′-CACGTG-3′) in the murine Tug1 promoter region 21 nt downstream of the ChoRE (Fig. 4*B*). We thought this E-box could be important because it is a consensus binding sequence for basic helix-loop-helix-leucine-zipper-containing transcription factors, such as MAX and MXD1 (35, 36). Thus, we first tested the role of this E-box on Tug1 suppression by generating the Tug1 promoter-luciferase reporters that contained a mutation in the E-box region (E-box mut) or a deletion of the E-box (ΔE-box). Both E-box mutants showed higher luciferase activities than the WT control (Fig. 4*C*), and the mutant activities were very similar to the promoter activity of the ChoRE mutant, suggesting that ChoRE and E-box function as a single *cis*-regulatory element, recruiting a cohort of transcription factors that downregulate Tug1 transcription.

Because it is well established that E-boxes recruit heterodimeric MAX and MXD1 transcription factors (35), we next examined the effect of these two transcription factors on Tug1 promoter activity. As shown in Fig. 4*D*, both Max and Mxd1 dose-dependently suppressed Tug1 promoter activity. Furthermore, because MXD1 has been shown to recruit HDAC1 to suppress gene transcription (37), we also performed ChIP assays using an HDAC1 antibody followed by qRT-PCR. We showed that under HG conditions, there was a marked enrichment of HDAC1 binding to the Tug1 promoter in cultured podocytes compared with that of the NG condition (Fig. 4*E*). Similarly, ChIP-qPCR also indicated enrichment of ChREBP, MLX, MAX, and MXD1 on the Tug1 promoter region (Fig. 4*E*). Using an HDAC1 inhibitor, trichostatin A (TSA), in cultured podocytes transfected with the Tug1-1kb promoter-luciferase construct, we tested whether inhibition of the HDAC1 resulted in a change in Tug1 promoter activity. We observed that TSA dose-dependently increased Tug1 promoter activity (Fig. 4*F*), suggesting that HDAC1 activity suppresses Tug1 expression. We also observed that a functional E-box in the Tug1-1kb promoter is necessary for this suppression, because the promoter lacking an intact E-box is unresponsive to TSA treatment (Fig. 4*F*).

**Figure 5.**
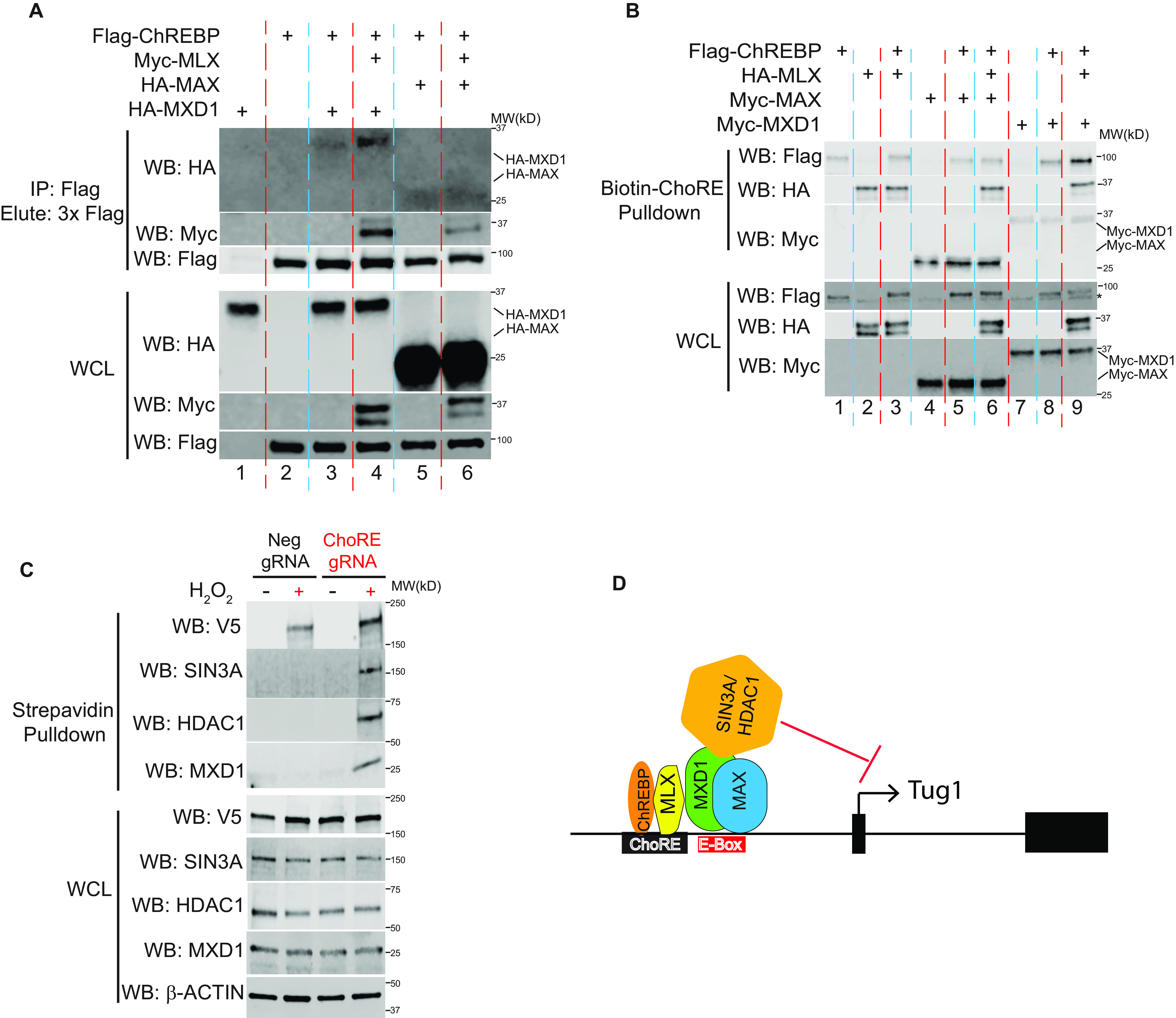
**ChREBP, MLX, MXD1, and HDAC1 form a complex in Tug1 promoter region in response to high glucose.** *A*, podocytes were transfected with the indicated plasmids, followed by Co-IP with anti-Flag M2 beads and elution with 3× Flag peptide. *Upper*, ChREBP-bound proteins, including MXD1, MAX, and MLX, were detected with immunoblots against HA and Myc antibody. *Lower*, expression of transfected plasmids in WCL. *B*, MLX enhances the binding of ChREBP/MXD1 complex to biotinylated ChoRE oligonucleotide. Podocytes were transfected with indicated plasmids, followed by biotin-ChoRE oligonucleotide pulldown with streptavidin beads. *Upper*, ChoRE oligo-bound proteins, including ChREBP, MXD1, MAX, and Mlx, were detected with immunoblots against Flag, HA, and Myc antibody, respectively. *Lower*, expression of transfected plasmids in WCL. An *asterisk* denotes a nonspecific band in the Flag blot. *C*, gRNA targeting ChoRE element in the Tug1 promoter induces APEX2-mediated biotinylation of MXD1, HDAC1, and SIN3A under high glucose. Immunoblots of streptavidin pulldown or WCL from proximity labeling of stable cells expressing the indicated gRNA and the dCas9-V5-APEX2 fusion protein cultured under high glucose with or without H_2_O_2_ treatment. *D*, proposed model of Tug1 transcription repression under high-glucose conditions in kidney podocytes. High glucose activates ChREBP/Mlx complexes that bind to the ChoRE motif in the Tug1 promoter, promoting the recruitment of MXD1 (and perhaps also MAX) to the adjacent E-box region. MXD1 further recruits an SIN3A/HDAC1 corepressor complex to suppress Tug1 transcription.

Further independent experiments were designed to interrogate the interactions of MAX/MXD1 with ChREBP/MLX on HG-mediated suppression of the Tug1 promoter. After generating several fusion constructs with short epitope tags, including ChREBP fused to a Flag tag, Mlx fused to a Myc tag, and Max and Mxd1 fused to HA tags, these constructs were then either individually or in various combinations cotransfected into the podocytes. Figure 5*A* shows the results of these experiments. The top three panels are the Western blotting results of the protein factors brought down by anti-Flag antibody, an indication of ChREBP-interacting proteins. The lower three panels exhibit the Western blotting results of the whole-cell lysate (WCL). From the anti-Flag immunoprecipitated proteins, we detected MXD1 (*lane 3*), MXD1 and MLX (*lane 4*), MAX (*lane 5*), and MAX and MLX (*lane 6*), suggesting that ChREBP interacts with MLX, MXD1, and MAX. Importantly, MLX can significantly enhance the interaction between MXD1 and ChREBP (*lane 4 versus lane 3*) but does not increase the association of MAX with ChREBP, suggesting that there is a ternary complex of ChREBP/MLX/MXD1.

To explore whether these transcription factors interact with each other in the context of the Tug1/ChoRE transcription, we performed a biotin-oligonucleotide pulldown assay, where a biotin-labeled ChoRE oligonucleotide from the Tug1 promoter region was incubated *in vitro* with cell extracts from podocytes transfected with combinations of these transcription factors (Fig. 5*B*). ChoRE DNA-bound proteins were then enriched by magnetic streptavidin beads and detected by immunoblotting. Whereas the bindings of MLX, MAX, and MXD1 to ChoRE DNA were easily detected, the binding of ChREBP to ChoRE was relatively weak but could be enhanced by the coexpression of MLX and MXD1 (*lane 9 versus lane 8*) but not MLX and MAX (*lane 6 versus lane 5*), suggesting that the presence of MLX/MXD1 further amplifies the binding of ChREBP to ChoRE on the Tug1 promoter.

Finally, we used CRISPR coupled with APEX2 proximity labeling to dissect transcription factors binding to the ChoRE region of the Tug1 gene under HG conditions. The gRNA targeting site is 12 bp from the ChoRE motif itself (Fig. 2*F*) and, therefore, falls within the expected ∼20-nm radius of APEX2 labeling. Our initial results indicated that ChREBP was labeled by APEX2 in a ChoRE gRNA-dependent manner (Fig. 2*G*). A follow-up APEX2 proximity labeling detected additional proteins, including MXD1, HDAC1, and SIN3A (Fig. 5*C*). This labeling was specific because it was not observed when nontargeting gRNA was used or H_2_O_2_ treatment was omitted. As a control, V5-tagged dCas9-APEX2 fusion protein was labeled by both gRNAs at comparable levels. Taken together, these data strongly indicate that under HG conditions, binding of the ChREBP/MLX heterodimer to ChoRE triggers the formation of a multisubunit complex, composed of at least MXD1, HDAC1, and SIN3A, to repress the transcription of Tug1 in podocytes.

A model depicting transcriptional regulation of HG-induced Tug1 expression, focusing on the roles of ChREBP, MLX, MXD1, and HDAC1, is shown in Fig. 5*D*.

## Discussion

Tug1 was first identified as an lncRNA transcript that could be upregulated by taurine in developing retinal cells (38). Despite the importance of Tug1 in cell homeostasis and its critical role in a number of unique biological activities, including kidney pathobiology and reproduction, information regarding the Tug1 promoter regulation is very limited; thus, the characterization of the Tug1 promoter is significant and provides insights into the underlying regulatory mechanisms of lncRNAs in general. To the best of our knowledge, this is the first report of transcriptional regulation of an lncRNA in response to glucose, and Tug1 is the first *bona fide* lncRNA transcriptional target gene of ChREBP in the literature.

Previous studies from our laboratory have demonstrated that Tug1 is markedly downregulated in response to HG (6). However, how HG milieu regulates the expression of Tug1 remained elusive. The current study outlines a mechanism coupling HG environment with the transcriptional repression of Tug1 in podocytes.

A network of multiple regulatory layers controls Tug1 gene expression. For instance, recently published work has shown that Tug1 expression can be induced by antioxidant taurine (38), hypoxia (39), TGF-β (40), and p53 (32, 33) but inhibited by polycomb repressive complex 2 (PRC2) (32, 33). In this study, we uncovered the unexpected regulatory effect of ChREBP on Tug1 expression and found that the effect of HG on Tug1 expression is, at least in part, transcriptional and through binding of ChREBP to the promoter region of Tug1.

Genome-wide ChIP-Seq studies from us and others have shown that ChREBP can bind to ChoRE, a carbohydrate response element, in the promoter of its target genes (20, 21). It has been proposed that under HG conditions, ChREBP-α is translocated from the cytoplasm into the nucleus, where it forms a heterodimer with MLX and binds to ChoRE motifs in the promoters of glycolytic and lipogenic genes to activate transcription (11, 14–16). Further studies have shown that glucose metabolites, such as glucose-6-phosphate, and posttranslational modifications, such as *O*-GlcNacylation, can also mediate ChREBP activation and its nuclear translocation, whereas adenosine monophosphate (AMP), ketone bodies, and cAMP suppress its activation (41–45). Interestingly, the suppression of ChREBP stimulates mitochondrial respiration, suggesting that ChREBP also plays a key role in redirecting glucose metabolism from oxidative phosphorylation pathways to glycolic pathways (46). This could be of significance, because in a previous study, we demonstrated that Tug1 upregulates a key transcription factor of mitochondrial biogenesis, PGC-1α (6). Thus, it is tempting to speculate that the suppression of Tug1 by ChREBP under HG conditions could contribute to the ChREBP-mediated effects on mitochondria. However, further studies are needed to explore these presumably mitochondrion-specific effects of ChREBP.

Our *in silico* and *in vitro* analyses have led to the identification of a regulatory repressor complex that interacts with ChoRE in the Tug1 promoter region. Indeed, our data indicate that along with ChREBP, a number of other factors, including MLX, MXD1, and HDAC1, play crucial roles in the HG-dependent Tug1 transcriptional regulation (Fig. 5*D*). Our data suggest that these transcription factors and cofactors interact with ChREBP, leading to Tug1 suppression. The current understanding of the mechanism by which ChREBP regulates gene transcription is that ChREBP dimerizes with the cofactor MLX and directly binds to ChoRE consensus sequences in target gene promoters (12, 13). Using peroxidase proximity labeling to tag proteins that are located in the vicinity of the ChoRE in the Tug1 promoter, we found ChREBP is a protein that was biotinylated. Our data also demonstrated that along with ChREBP, other transcription factors, including MXD1 as well as HDAC1, are enriched at the Tug1 promoter, suggesting that histone deacetylation is important in HG-mediated Tug1 repression. It has been previously reported that the MXD family of proteins dimerize with MAX and recruit a histone deacetylase complex through an N-terminal Sin3 interaction domain, thereby suppressing transcription (37, 47–50). Importantly, the involvement of MXD1 and HDAC1 in the suppression of target genes was previously described in the context of cancer progression (51). However, the role of these factors in HG-mediated repression of Tug1 expression was unknown.

### Conclusions

Our observations demonstrate that the transcription of the lncRNA Tug1 is suppressed under HG conditions in podocytes. We characterized the promoter region for Tug1 transcription and identified ChREBP and its partner, MLX, as transcriptional factors that bind to a ChoRE motif in the promoter. Our results also provide evidence that, along with ChREBP and its obligate partner, MLX, other key factors, including MXD1 and HDAC1, coordinate the regulatory effect of HG on the Tug1 promoter. Thus, our studies further strengthen the relevance of Tug1 and ChREBP in podocyte response to HG and provide strong evidence for considering Tug1 and ChREBP as potential targets for novel diabetic nephropathy therapy. Furthermore, we demonstrate that ChREBP coordinates with MLX, MXD1, and HDAC1 to suppress the Tug1 promoter. In this regard, our results establish a basis for future efforts to identify regulatory factors and mechanisms involved in the transcription of other lncRNAs in podocytes.

## Experimental procedures

### Tissue culture

Conditionally immortalized mouse podocytes were cultured as previously reported (52). Briefly, podocytes were cultured on BD BioCoat collagen I plates (BD Biosciences, San Jose, CA) at 33 °C in RPMI 1640 complete medium with 20 units/ml mouse recombinant IFN-γ (Thermo Fischer, Carlsbad, CA). To induce differentiation, podocytes were cultured in DMEM (5 mm glucose and 5% FBS) at 37 °C without IFN-γ for 10–12 days. For glucose treatment experiments, podocytes were serum deprived for 24 h prior to addition of normal glucose (NG; 5 mm) or high glucose (HG; 25 mm). Mouse renal tubular epithelial cells (TCMK-1; CCL-139), human cervical carcinoma HeLa cells (CCL-6), and human embryonic kidney fibroblast 293T cells (CRL-3216) all were obtained from the ATCC and cultured according to the instructions at 37 °C. All cell culture experiments were repeated at least three independent times. To assess the stability of Tug1 RNA, cells were treated with actinomycin D (4 mm; MilliporeSigma, St. Louis, MO) or with vehicle (DMSO) added into cells to 4 mm.

### Animal work

All animal studies were conducted according to the Principles of Laboratory Animal Care (NIH publication no. 85023, revised 1985) and the guidelines of the IACUC of The University of Texas MD Anderson Cancer Center. Diabetic db/db mice and their control littermates, db/m, were obtained from Jackson Laboratories (strain 000642, BKS.Cg-Dock7^m+/+^Lepr^db/J^; Bar Harbor, ME). All mice used in experiments were male. No animals were excluded from the studies performed. All animals were maintained on a normal chow diet and housed in a room with a 12-h/12-h light/dark cycle and an ambient temperature of 22 °C. Kidney podocytes were isolated by positive selection with biotin-labeled Kirrel3 and podocalyxin antibodies (2.5 μg/antibody/mouse; R&D Systems, Minneapolis, MN) followed by Dynabeads M-450, as previously described (53).

### RNA extraction and real-time RT-PCR

Total RNAs were extracted using the PureLink RNA Mini Kit (Thermo Fischer Scientific) with on-column digestion of DNase I (New England Biolabs, Ipswich, MA). After reverse transcription using an iScript cDNA synthesis kit (Bio-Rad, Hercules, CA), cDNAs were diluted into 10 ng per well and quantified by real-time PCR using PowerUp SYBR Green master mix (Thermo Fischer) on a StepOnePlus real-time PCR system (Applied Biosystems). Individual samples were run in duplicate, and each experiment was repeated at least 3 times. Relative gene expression was calculated using the 2^−ρCT^ method (54). Sequences of gene-specific primers are listed in Table S1.

### Transcription factor binding site analysis, subcloning, mutagenesis, luciferase assay, and modulation of ChREBP expression

Sequences of putative 1-kb promoters of the *TUG1* gene from human or *Tug1* genes from mouse and rat were retrieved from the UCSC Genome Browser as 1 kb of genomic DNA upstream of each transcription start site (TSS) and aligned to mouse *Tug1* promoter using Clustal Omega (RRID:SCR_001591). Binding sites for transcription factors were analyzed using rVista 2.0 (RRID:SCR_018707). Expression plasmids of Flag-YY1 (Addgene, 104396), Myc-ChREBP-α (CA-ChREBP) (55), Myc-ChREBP (55), Myc-Mlx (15), and Myc-DN-Mlx (dominant negative) (15) in the pcDNA3 backbone or p53 (Addgene, 16434) and Flag-HDAC1 (56) in the pCMV5 vector were previously described. Mouse ChREBP cDNA (55), mouse Mlx cDNA (15), human MAX cDNA (Addgene, 82944), and MXD1 cDNA (human ORFeome clone ID 100072779, from Dharmacon, provided by the Functional Genomics Core at MDACC) were also subcloned by PCR using appropriate primers (see Table S1 for detailed sequence) into modified pRK5 vectors with a Flag tag or a 3× HA tag at the N terminus (57) or into the pCS3-6Myc vector (56) with a 6× Myc tag at the N terminus to generate Flag-ChREBP, HA-Mlx, HA-MAX, HA-MXD1, Myc-MAX, and Myc-MXD1. The mouse *Tug1* gene 1-kb proximal promoter region (−996 to −1) was amplified from cultured mouse podocyte genomic DNA by PCR using Herculase II fusion DNA polymerase (Agilent, Santa Clara, CA) with the primers CAGGTACCAAACACAGCTTGCTATTATGCC (forward) TCCTCGAGCTGCGCCCCAAGAGCTGGAT (reverse) and subcloned into a KpnI-XhoI digest site of the promoterless luciferase reporter vector pGL4.10 [luc2] (Promega, Madison, WI). Site-directed mutagenesis was carried out using a QuikChange II site-directed mutagenesis kit (Agilent). The putative ChREBP binding sites ChoRE1 (CACGTGACCGGATCTTG, −324 to −308) and ChoRE2 (TCCGCCCCCATCACGTG, −298 to −282) were mutated into CACGTGACGGATCTTG and TCCGCCCCATCACGTG, respectively, where the 5-nt spacer between the two E-boxes in ChoRE motifs were shortened to 4 nt (underlined) as previous studies showed (10, 34). E-box mutants were designed so the E-box in ChoRE2, GCCCCCATCACGTGAGGGCGCG, was mutated into a non-E-box, GCCCCCATTTACAAGGGCGCG, or completely deleted into GCCCCCATAGGGCGCG. All constructs were verified by sequencing.

For experiments using the pGL4.10 luciferase reporter constructs *in vitro*, 1.5 × 10^5^ undifferentiated podocytes were plated in 12-well plates and transfected the next morning with Lipofectamine 2000 according to the manufacturer's instructions. 24 h posttransfection, cells were serum starved overnight and treated with glucose or with trichostatin A (MilliporeSigma), if necessary, for 24 h before harvest, as previously described (37, 50, 58). Luciferase activity was measured using the Steady-Glo luciferase assay system (Promega, Madison, WI) on a BioTek Synergy 2 microplate reader with luminescence normalized to β-galactosidase.

Glycerol stocks of GIPZ-based lentiviral shRNAs against mouse ChREBP or a nonsilencing control (see Table S1 for detailed sequence) (from Dharmacon) were provided by the Functional Genomics Core at MDACC. Lentiviral plasmids were transfected into HEK 293T cells together with packaging plasmids psPAX2 and pMD2.G (Addgene, Watertown, MA) using Lipofectamine 2000 (Thermo Fischer). Lentiviruses concentrated with Lenti-X Concentrator (Clontech, Mountain View, CA) were used to infect undifferentiated podocytes in the presence of 6 μg/ml Polybrene (MilliporeSigma). After selection with 1 μg/ml puromycin and FACS sorting via the GFP channel in 1 cell/well onto a 96-well plate by the Flow Cytometry and Cellular Imaging Core Facility at MDACC, single stable clones were collected. For gain-of-function study of ChREBP, mouse ChREBP cDNA (55) was subcloned into the Zeo-pT-MCS-GFP-T2A-Puro plasmid (28) and transfected together with *piggyBac* transposase (29) into undifferentiated podocytes. Single stable clones were collected as above after puromycin selection and FACS sorting.

### CRISPR/APEX-mediated proximity labeling and streptavidin pulldown

We engineered the dCas9-APEX2-NLS construct from two Addgene clones (97421 [24] and 124617 [27]), and the expression cassette was introduced into a Zeo-pT-MCS-GFP-T2A-Puro *piggyBac* expression vector (28); gRNAs against the mouse *Tug1* promoter region or a scramble control (sequence in Table S1) were cloned into the BsaI sites of pX333 (Addgene, 64073), released with XbaI-SnaB double digestion, and cloned into the same piggyBac-dCas9-APEX2-NLS vector. Constructs were verified by sequencing (see Fig. S2B for complete sequence) and introduced into podocytes together with *piggyBac* transposase (29) and selected with 1 μg/ml puromycin. Proximity labeling was performed as recently described (24, 30, 59). Briefly, cells at 80–90% confluency were pretreated with 500 μm biotin-tyramide (Iris Biotech Gmbh) for 30 min, followed by 1 mm H_2_O_2_ for 1 min. After quenching and washing three times with DBPS with 5 mm Trolox and 10 mm sodium ascorbate, cells were scraped into PBS. Cell pellets were lysed in RIPA buffer, 1 mm PMSF, 5 mm Trolox, 10 mm sodium ascorbate, and 10 mm sodium azide. Cell lysates were incubated with prewashed Pierce streptavidin magnetic beads (Thermo Fischer) overnight in a cold room. The beads were washed twice with RIPA buffer and once each with 1 m KCl, 0.1 m Na_2_CO_3_, and 2 m urea in 10 mm Tris-HCl (pH 8.0) and twice with RIPA buffer. Bound biotinylated proteins were eluted in 1× SDS sample buffer (Bio-Rad) and separated on 4–20% PAGE (Bio-Rad), followed by immunoblotting with antibodies against ChREBP, Mxd1, Sin3A, HDAC1, V5, and β-actin.

### Immunoprecipitation, biotinylated oligonucleotide pulldown assay, and ChIP-qPCR

Co-IPs were performed in transiently transfected podocytes or HEK293T cells as previously described (56). Briefly, cells were lysed in TNMG buffer (50 mm Tris-HCl, pH 8.0, 50 mm NaCl, 5 mm MgCl_2_, 10% glycerol, 0.5% Nonidet P-40) followed by IP with magnetic anti-Flag M2 beads (MilliporeSigma), eluted with 100 μg/ml 3× Flag peptide (MilliporeSigma) and then boiled in 1× SDS sample buffer, followed by immunoblotting.

Biotinylated oligonucleotide pulldown assay was carried out essentially as previously described (58, 60). Briefly, podocytes were transiently transfected with the indicated plasmids and collected in S1 lysis buffer (10 mm HEPES, pH 7.5, 150 mm NaCl, 1 mm MgCl_2_, 0.5 mm EDTA, 0.5 mm DTT, 0.1% NP-40, 10% glycerol). The cell lysates were incubated with 40 pmol biotinylated ChoRE oligonucleotides and 5 μg of poly(dI-dC) (Santa Cruz) at 4 °C for 16 h. DNA-bound proteins were collected with Dynabeads M-280 (Thermo Fischer) for 2 h and washed 5 times with S1 lysis buffer, followed by immunoblotting. Validated tag-specific antibodies were used: anti-Flag (SigmaMillipore, F1804, and Cell Signaling, 2368), anti-HA (Cell Signaling, 3724), anti-Myc (Cell Signaling, 2276), and anti-V5 (Cell Signaling, 13202). Western blots were imaged on an Odyssey FC imaging system (Li-Cor, Lincoln, NE) using appropriate DyLight fluorescent secondary antibodies (Thermo Fischer) and were analyzed and quantitated using Image Studio Lite v5.25 (Li-Cor).

ChIP was carried out using a SimpleChIP enzymatic chromatin IP kit (Cell Signaling) according to the manufacturer's instructions, where chromatin was partially digested with Micrococcal Nuclease (New England Biolabs) to ensure nucleosome release. Validated ChIP-grade antibodies were used: anti-ChREBP (Novus, NB400-135), anti-MLX (Cell Signaling, 85570), anti-MAX (Proteintech, 10426-1-AP), anti-MXD1 (Proteintech, 19547-1-AP), anti-HDAC1 (Active Motif, 91215), anti-SIN3A (Active Motif, 39865), or normal rabbit IgG (Cell Signaling, 2729). Purified chromatin was quantitated by real-time qPCR using specific primers spanning the ChREBP binding elements in the *Tug1* promoter (see Table S1 for sequence). The amount of immunoprecipitated DNA in each sample is represented as fold of enrichment relative to the input chromatin.

### Statistical analysis

Group data are expressed as mean ± S.E.M. Comparisons of multiple groups were performed using one-way analysis of variance followed by Tukey's multiple-comparisons test. Comparisons between two groups were performed using Student's *t* test. All tests were two-tailed, with a *p* value of <0.05 considered a statistically significant result. Tests were performed with Prism ver. 8.0 (GraphPad, San Diego, CA).

## Data availability

All data are contained within this manuscript.

## Supplementary Material

Supporting Information
