# Supplementary material for: Role for carbohydrate response element-binding protein (ChREBP) in high glucose-mediated repression of long noncoding RNA Tug1
Source: J Biol Chem. 2020 May 28;295(47):15840–52. doi: 10.1074/jbc.RA120.013228 (PMC7681008; doi:10.1074/jbc.RA120.013228)
Supplement: Supporting Information [file supp_295_47_15840__index.html]

Role for carbohydrate response element-binding protein (ChREBP) in high glucose-mediated repression of long noncoding RNA Tug1 — Tug1 transcription regulation by ChREBP — Role for carbohydrate response element-binding protein (ChREBP) in high glucose-mediated repression of long noncoding RNA Tug1 — Tug1 transcription regulation by ChREBP — Supporting Information 

# Role for carbohydrate response element-binding protein (ChREBP) in high glucose-mediated repression of long noncoding RNA Tug1

## Supporting Information

- Supporting Information (to be published online) - One Table and three Figures
