# Supplementary material for: Role for carbohydrate response element-binding protein (ChREBP) in high glucose-mediated repression of long noncoding RNA Tug1
Source: J Biol Chem. 2020 May 28;295(47):15840–52. doi: 10.1074/jbc.RA120.013228 (PMC7681008; doi:10.1074/jbc.RA120.013228)
Supplement: Supporting Information [file supp_RA120.013228_159108_2_supp_534182_qwnj61.pdf]

Supporting Table Oligonucleotide Sequences

PCR cloning primers

Gene Name	Forward primer(5'-3')	Reverse primer (5'-3')
Tug1-1kb promoter (mouse)	CAGGTACCAAACACAGCTTGCTATTATGCC	TCCTCGAGCTGCGCCCCAAGAGCTGGAT
Mlx–β (mouse)	tcgaattcACGGAGCCGGGCGCCTCTCC	ctCTCGAGTCAGTAGAGTTGGTTTTTC
MAX (human)	tgGAATTCagcgataacgatgacatcga	agGTCGACtcagctggcctccatccgga
MXD1 (human)	tgGAATTCgcggcggcggttcggatgaa	gtTCTAGAttagagaccaagacacgccttgtgactgtcct

qPCR primers

Gene Name	Forward primer(5'-3')	Reverse primer (5'-3')
Gapdh	GCCTGGAGAAACCTGCCAA	CGAAGGTGGAAGAGTGGGAG
Actb	CTAAGGCCAACCGTGAAAAG	ACCAGAGGCATACAGGGACA
Chrebp (Mlxipl)(total)	CACTCAGGGAATACACGCCTAC	ATCTTGGTCTTAGGGTCTTCAGG
Chrebp-α	CGACACTCACCCACCTCTTC	TTGTTCAGCCGGATCTTGTC
Chrebp-β	TCTGCAGATCGCGTGGAG	CTTGTCCCGGCATAGCAAC
Morc2a	AGGAGCCATTTCCTGAGTCAACCA	ACACCTGCACACCCTGAGAATCTT
Tug1-a/b/c	TCCAAAGTGAACTACGTCCCG	TGGGCAGAGTAGGACAGGAG
Tug1-a	CGTCTTTCTTCTCGGCCAGA	CTCCTTCTCCAGAGGAAAGC
Tug1-b	CTTCTGACTCCCAGTCCAGT	ATCATCAACCACCACGGAGA
Tug1-a/c	CATAGTATCATCTTCGGGTTAC	CACAAAATGCATGTAGGTTC
Tug1-b/c	CAAATGTGAGACTGAATGGCCA	TGCTTAGAGAGCTGTACCACT
Pgc1a(Ppargc1a)	TCCTCTTCAAGATCCTGTTAC	CACATACAAGGGAGAATTGC
GAPDH	GGTGGTCTCCTCTGACTTCAACA	GTTGCTGTAGCCAAATTCGTTGT
ACTB	CTGTGGCATCCACGAAACTA	AGGAAAGACACCCACCTTGA
ChREBP (MLXIPL)	AGTGCTTGAGCCTGGCCTAC	TTGTTCAGGCGGATCTTGTC
MORC2	CCCAAGCAGACCTGAAGAAA	TTCCTGATCACAGCAGGTAAAG
TUG1	AGTGAATTATGTCCTGTGCCT	GATGGGTGAATGCCTCCTG

ChIP-qPCR primers

Name	Forward primer(5'-3')	Reverse primer (5'-3')
Tug1	AGCGCACCTTCGGCCAATCACA	AATGGCCGAGAGGGGTCAGGAA
Neg control	GTGACCTAGGTTCCAGATTC	GCAGAGTAGAGACCATAGAC

gRNAs targeting Tug1

Name	sequence (PAM sequence highlighted)	targeting strand/region
ChoRE-gRNA	GACGTCGCTCGCCAATCAGA**AGG**	antisense/ChoRE
non-targeting control	GCGAGGTATTCGGCTCCGCG**NGG**	non-targeting

shRNA for ChREBP knockdown

Name	target (antisense)
shChREBP	TGAAGTTCTTCCACTTGGG
non-silencing control	TTACTCTCGCCCAAGCGAG

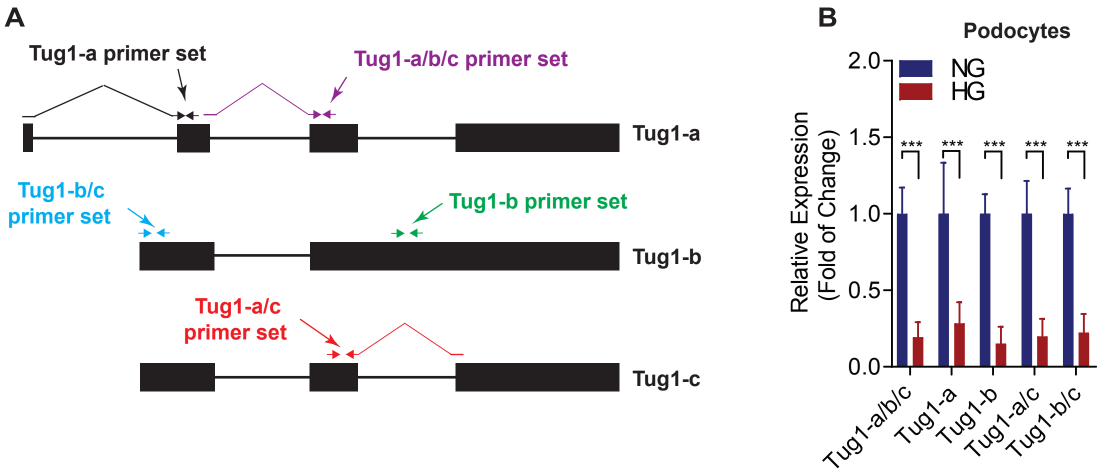

Supporting Figure 1. Response of *Tug1* isoforms to high glucose milieu in cultured podocytes. *A,* Three isoforms of the murine Tug1 transcripts: -a, -b and -c with their corresponding genomic structures. Black boxes represent exons, and lines represent introns. Primer sets to identify different isoform(s) by RT-qPCR are indicated and color-coded. Tug1-a forward, Tug1-a/b/c forward, and Tug1-a/c reverse primers are designed to anneal across the junction of two neighnoring exons. *B*, Quantitative analysis of the Tug1-isoforms in mouse podocytes cultured under normal glucose (NG, 5mM) or high glucose (HG, 25mM) conditions. Cells were cultured for 48 hrs under NG (5 mM) or HG (25 mM) conditions, RNAs were extracted and analyzed by RT-qPCR (n=3). Expression values were normalized to Gapdh. Data are presented as mean±s.e.m. ***p<0.001.

A

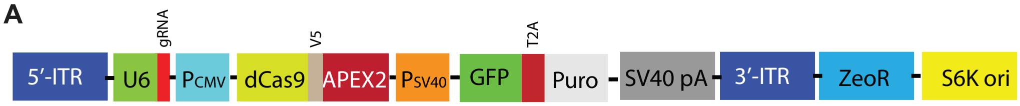

B

```
>Sequence of Zeo-pT-U6-ChoREgRNA-dCas9-APEX2-GTP
GAATTCTTAACCCTAGAAAGATAGTCTGCGTAAAATTGACGCATGCATTCTTGAAATATTGCTCTCTCTTTCTAAATAGCGCGAATCCGTCGCTGTGCATTTAGGACATCTCAGTCGCCGCTTGGAGCTCCCGTGAGGCGTGCTTGTCAATGCG
GTAAGTGTCACTGATTTTGAACTATAACGACCGCGTGAGTCAAAATGACGCATGATTATCTTTTACGTGACTTTTAAGATTTAACTCATACGATAATTATATTGTTATTTCATGTTCTACTTACGTGATAACTTATTATATATATATATTTTCTTG
TTATAGATACTCGACGGTACCCTCGAGAGATCTActagagagggcctatttcccatgattccttcatatttgcatatacgatacaaggctgttagagagataattggaattaatttgactgtaaacacaaagatattagtacaaaatacgtgac
gtagaaagtaataatttcttgggtagtttgcagttttaaaattatgttttaaaatggactatcatatgcttaccgtaacttgaaagtatttcgatttcttggctttatatatcttgtggaaaggacgaaaCACCGgacgtcgctcgccaatcag
agttttagagctagaaatagcaagttaaaataaggctagtccgttatcaacttgaaaaagtggcaccgagtcggtgctttttttgttttagagctagaaatagcaagttaaaataaggctagtccgtttttagcgcgtgcgccaattctgcagac
aaatggggtacccgttacataacttacggtaaatggcccgcctggctgaccgcccaacgacccccgcccattgacgtcaatagtaacgccaatagggactttccattgacgtcaatgggtggagtatttacggtaaactgcccacttggcagta
catcaagtgtatcatatgccaagtacgccccctattgacgtcaatgacggtaaatggcccgcctggcattgtgcccagtacatgaccttatgggactttcctacttggcagtacatctacgtattagtcatcgctattaccatggtgatgcggt
tttggcagtacatcaatgggcgtggatagcggtttgactcacggggatttccaagtctccaccccattgacgtcaatgggagtttgttttggcaccaaaatcaacgggactttccaaaatgtcgtaacaactccgccccattgacgcaaatggg
cggtaggcgtgtacggtgggaggtctatataagcagagctctctggctaactagagaacccactgcttactggcttatcgaaattaatacgactcactatagggagacccaagcttgcggccctagccaccatggactacaaagaccatgacgg
tgattataaagatcatgacatcgattacaaggatgacgatgacaagatggccccaagaagaagaggaaggtgggccgcggaatggacaagaagtactccattgggctcgccatcggcacaaacagcgtcggctgggccgtcattacggacgag
tacaaggtgccgagcaaaaaattcaaagttctgggcaataccgatcgccacagcataaagaagaacctcattggcgccctcctgttcgactccggggaaaccgccgaagccacgcggctcaaaagaacagcacggcgcagatatacccgcagaa
agaatcggatctgctacctgcaggagatctttagtaatgagatggctaaggtggatgactctttcttccataggctggaggagtcctttttggtggaggaggataaaaagcacgagcgccacccaatctttggcaatatcgtggacgaggtggc
gtaccatgaaaagtacccaaccatatatcatctgaggaagaagcttgtagacagtactgataaggctgacttgcggttgatctatctcgcgctggcgcatatgatcaaatttcggggacacttcctcatcgaggggggacctgaacccagacaac
agcgatgtcgacaaactctttatccaactggttcagacttacaatcagcttttcgaagagaacccgatcaacgcatccggagttgacgccaaagcaatcctgagcgctaggctgtccaaatcccggcggctcgaaaacctcatcgcacagctcc
ctggggagaagaagaacggcctgtttggtaatcttatcgccctgtcactcgggctgacccccaactttaaatctaacttcgacctggccgaagatgccaagcttcaactgagcaaagacacctacgatgatgatctcgacaatctgctggccca
gatcggcgaccagtacgcagaccttttttggcggcaaagaacctgtcagacgccattctgctgagtgatattctgcgagtgaacacggagatcaccaaagctccgctgagcgctagtatgatcaagcgctatgatgagcaccaccaagacttg
actttgctgaaggcccttgtcagacagcaactgcctgagaagtacaaggaaattttcttcgatcagtctaaaaatggctacgccggatacattgacggcggagcaagccaggaggaattttacaaatttattaagcccatcttggaaaaaatgg
acggcaccgaggagctgctggtaaagcttaacagagaagatctgttgcgcaaacagcgcactttcgacaatggaagcatccccaccagattcacctgggcgaactgcacgctatcctcaggcggcaagaggatttctacccctttttgaaaga
taacagggaaaagattgagaaaatcctcacatttcggataccctactatgtaggcccccctcgcccggggaaattccagattcgcgtggatgactcgcaaatcagaagagaccatcactccctggaacttcgaggaagtcgtggataaggggggcc
tctgcccagtccttcatcgaaaggatgactaactttgataaaaatctgcctaacgaaaaggtgcttcctaaacactctctgctgtacgagtacttcacagtttataacgagctcaccaaggtcaaatacgtcacagaaggatgagaaagccag
cattcctgtctggagagcagaagaaagctatcgtggacctcctcttcaagacgaaccggaaagttaccgtgaaacagctcaaagaagactatttcaaaaagattgaatgtttcgactctgttgaaatcagcggagtggaggatcgcttcaacgc
atccctgggaacgtatcacgatctcctgaaaatcattaaagacaaggacttcctggacaatgaggagaacgaggacattcttgaggacattgtcctcacccttacgttgtttgaagatagggagatgattgaagaacgcttgaaaacttacgct
catctcttcgacgacaaagtcatgaaacagctcaagaggcgccgatatacaggatgggggcggctgtcaagaaaactgatcaatgggatccgagacaagcagagtggaaagacaatcctggattttcttaagtccgatggatttgccaaccgga
acttcatgcagttgatccatgatgactctctcacctttaaggaggacatccagaaagcacaagtttctggccaggggggacagtcttcacgagcacatcgctaatcttgcaggtagcccagctatcaaaaagggaatactgcagaccgttaaggt
cgtggatgaactcgtcaaagtaatgggaaggcataagcccgagaatatcgttatcgagatggcccgagagaaccaaactacccagaagggacagaagaacagtagggaaaggatgaagaggattgaagagggtataaaagaactggggtcccaa
atccttaaggaacacccagttgaaaacacccagcttcagaatgagaagctctacctgtactacctgcagaacggcagggacatgtacgtggatcaggaactggacatcaatcggctctccgactacgacgtggatgccatcgtgccccagtctt
ttctcaaagatgattctattgataataaagtgttgacaagatccgataaaaatagagggaagagtgataacgtcccctcagaagaagttgtcaagaaaatgaaaaattattggcggcagctgctgaacgccaaactgatcacacaacggaagtt
cgataatctgactaaggctgaacgaggtggcctgtctgagttggataaagccggcttcatcaaaaggcagcttgttgagacacgccagatcaccaagcacgtggcccaaattctcgattcacgcatgaacaccaagtacgatgaaaatgacaaa
ctgattcgagaggtgaaagttattactctgaagtctaagctggtctcagatttcagaaaggactttcagttttataaggtgagagagatcaacaattaccaccatgcgcatgatgcctacctgaatgcagtggtaggcactgcacttatcaaaa
aatatcccaagcttgaatctgaatttgtttacggagactataaagtgtacgatgttaggaaaatgatcgcaaagtctgagcaggaaataggcaaggccaccgctaagtacttcttttacagcaatattatgaatttttttcaagaccgagattac
actggccaatggagagattcggaagcgaccacttatcgaaacaaacggagaaacaggagaaatcgtgtgggacaagggtagggatttcgcgacagtccggaaggtcctgtccatgccgcaggtgaacatcgttaaaaagaccgaagtacagacc
ggaggcttctccaaggaaagtatcctcccgaaaaggaacagcgacaagctgatcgcacgcaaaaaagattgggaccccaagaaatacggcggattcgattctcctacagtcgcttacagtgtactggttgtggccaaagtggagaaagggaagt
ctaaaaaactcaaaagcgtcaaggaactgctgggcatcacaatcatggagcgatcaagcttcgaaaaaaaccccatcgactttctcgaggcgaaaggatataaagaggtcaaaaaagacctcatcattaagcttcccaagtactctctctttga
gcttgaaaacggccggaaacgaatgctcgctagtgcgggcgagctgcagaaaggtaacgagctggcactgccctctaaatacgttaatttcttgtatctggccagccactatgaaaagctcaaagggtctcccgaagataatgagcagaagcag
ctgttcgtggaacaacacaaacactaccttgatgagatcatcgagcaaataagcgaattctccaaaagagtgatcctcgccgacgctaacctcgataaggtgctttctgcttacaataagcacagggataagcccatcagggagcaggcagaaa
acattatccacttgtttactctgaccaacttgggcgcgcctgcagccttcaagtacttcgacaccaccatagacagaaagcggtacacctctacaaaggaggtcctggacgccacactgattcatcagtcaattacggggctctatgaaacaag
aatcgacctctctcagctcggtggagacgtgcggggttctggcaagcccatccccaacccccctgctgggcctggacagcaccggaaagtcttacccaactgtgagtgctgattaccaggacgccgttgagaaggcgaagaagaagctcagaggc
ttcatcgctgagaagagatgcgctcctctaatgctccgtttggcattccactctgctggaacctttgacaagggcacgaagaccggtggacccttcggaaccatcaagcaccctgccgaactggctcacagcgctaacaacggtcttgacatcg
ctgttaggcttttggagccactcaaggcggagttccctattttgagctacgccgatttctaccagttggctggcgttgttgccgttgaggtcacgggtggacctaaggttccattccaccctggaagagaggacaagcctgagccaccaccaga
gggtcgcttgcctgatcccactaagggttctgaccatttgagagatgtgtttggcaaagctatggggcttactgaccaagatatcgttgctctatctggggggtcacactattggagctgcacacaaggagcgttctggatttgagggtccctgg
acctctaatcctcttatttcgacaactcatacttcacggagttgttgagtggtgagaaggaaggtctccttcagctaccttctgacaaggctcttttgtctgaccctgtattccgccctctcgttgataaatatgcagcggacgaagatgcct
tctttgctgattacgctgaggctcaccaaaagctttccgagcttgggtttgctgatgccgaattcagcagggccgaccccaagaagaagaggaaggtggaccccaagaagaagaggaaggtggaccccaagaagaagaggaaggtgtgactcga
gcatgcatctagagggccctattctatagtgtcacctaaatgctagagctcgctgatcagcctcgactgtgccttctagttgccagccatctgttgtttgcccctcccccgtgccttccttgaccctggaaggtgccactcccactgtcctttc
ctaataaaatgaggaaattgcatcgcattgtctgagtaggtgtcattctattctggggggtggggtggggcaggacagcaaggggggaggattgggaagacaatagcaggcatgctggggatgcggtgggctctatggcttctgaggcggaaaga
accagCTGTGGAATGTGTGTCAGTTAGGGTGTGGAAAGTCCCCAGGCTCCCCAGCAGGCAGAAGTATGCAAAGCATGCATCTCAATTAGTCAGCAACCAGGTGTGGAAAGTCCCCAGGCTCCCCAGCAGGCAGAAGTATGCAAAGCATGCATCT
CAATTAGTCAGCAACCATAGTCCCGCCCCTAACTCCGCCCATCCCGCCCCTAACTCCGCCCAGTTCCGCCCATTCTCCGCCCCATGGCTGACTAATTTTTTTTATTTATGCAGAGGCCGAGGCCGCCTCGGCCTCTGAGCTATTCCAGAAGTAG
TGAGGAGGCTTTTTTGGAGGCTAGCatggtgagcaagggcgaggagctgttcaccggggtggtgcccatcctggtcgagctggacggcgacgtaaacggccacaagttcagcgtgtccggcgagggcgagggcgatgccacctacggcaagctg
accctgaagttcatctgcaccaccggcaagctgcccgtgccctggcccaccctcgtgaccaccctgacctacggcgtgcagtgcttcagccgctaccccgaccacatgaagcagcacgacttcttcaagtccgccatgcccgaaggctacgtcc
aggagcgcaccatcttcttcaaggacgacggcaactacaagacccgcgccgaggtgaagttcgagggcgacaccctggtgaaccgcatcgagctgaagggcatcgacttcaaggaggacggcaacatcctggggcacaagctggagtacaacta
caacagccacaacgtctatatcatggccgacaagcagaagaacggcatcaaggtgaacttcaagatccgccacaacatcgaggacggcagcgtgcagctcgccgaccactaccagcagaacacccccatcggcgacggccccgtgctgctgccc
gacaaccactacctgagcacccagtccgccctgagcaaagaccccaacgagaagcgcgatcacatggtcctgctggagttcgtgaccgccgccgggatcactctcggcatggacgagctgtacaagtaCAGCGGTCGCGAGGGCAGAGGAAGTC
TTCTAACATGCGGTGACGTGGAGGAGAATCCCGGCCCTATGACCGAGTACAAGCCCACGGTGCGCCTCGCCACCCGCGACGACGTCCCCAGGGCCGTACGCACCCTCGCCGCCGCGTTCGCCGACTACCCCGCCACGCGCCACACCGTCGATCC
GGACCGCCACATCGAGCGGgtcaccgagctgcaagaactcttcctcacgcgcgtcgggctcgacatcggcaaggtgtgggtcgcggacgacggcgccgcggtggcggtctggaccacgccggagagcgtcgaagcgggggcggtgttcgccgag
atcggcccgcgcatggccgagttgagcggttcccggctggccgcgcagcaacagatggaaggcctcctggcgccgcaccggcccaaggagcccgcgtggttcctggccaccgtcggcgtctcgcccgaccaccagggcaagggtctgggcagcg
ccgtcgtgctccccggagtggaggcggccgagcgcgccggggtgcccgccttcctggagacctccgcgccccgcaacctccccttctacgagcggctcggcttcaccgtcaccgccgacgtcgaggtgcccgaaggaccgcgcacctggtgcat
gacccgcaagcccggtgcctgaGGCGCGGGCCGCGCTAGCGGATCCGCTTCGAGCAGACATGATAAGATACATTGATGAGTTTGGACAAACCACAACTAGAATGCAGTGAAAAAAATGCTTTATTTGTGAAATTTGTGATGCTATTGCTTTATT
TGTAACCATTATAAGCTGCAATAAACAAGTTAACAACAACAATTGCATTCATTTTATGTTTCAGGTTCAGGGGGAGATGTGGGAGGTTTTTTAAAGCAAGTAAAACCTCTACAAATGTGGTAAAATCCGATAAGGATCGATCCGGGCGGATCCT
TTTGTTACTTTATAGAAGAAATTTTGAGTTTTTGTTTTTTTTTAATAAATAAATAAACATAAATAAATTGTTTGTTGAATTTATTATTAGTATGTAAGTGTAAATATAATAAAACTTAATATCTATTCAAATTAATAAATAAACCTCGATATAC
AGACCGATAAAACACATGCGTCAATTTTACGCATGATTATCTTTAACGTACGTCACAATATGATTATCTTTCTAGGGTTAATCTAGAGTCGACAAAGGCTAAGTAACATCTGTGGCTTAATTAAAACAGTAGTTGACAATTAAACATTGGCATA
GTATATCTGCATAGTATAATACAACTCACTATAGGAGGGCCATCATGGCCAAGTTGACCAGTGCTGTCCCAGTGCTCACAGCCAGGGATGTGGCTGGAGCTGTTGAGTTCTGGACTGACAGGTTGGGGTTCTCCAGAGATTTTGTGGAGGATGA
CTTTGCAGGTGTGGTCAGAGATGATGTCACCCTGTTCATCTCAGCAGTCCAGGACCAGGTGGTGCCTGACAACACCCTGGCTTGGGTGTGGGTGAGAGGACTGGATGAGCTGTATGCTGAGTGGAGTGAGGTGGTCTCCACCAACTTCAGGGAT
GCCAGTGGCCCTGCCATGACAGAGATTGGAGAGCAGCCCTGGGGGAGAGAGTTTGCCCTGAGAGACCCAGCAGGCAACTGTGTGCACTTTGTGGCAGAGGAGCAGGACTGAGGATAACCTAGGAAACCTTAAAACCTTTAAAAGCCTTATATAT
TCTTTTTTTTCTTATAAAACTTAAAACCTTAGAGGCTATTTAAGTTGCTGATTTATATTAATTTTATTGTTCAAACATGAGAGCTTAGTACATGAAACATGAGAGCTTAGTACATTAGCCATGAGAGCTTAGTACATTAGCCATGAGGGTTTAG
TTCATTAAACATGAGAGCTTAGTACATTAAACATGAGAGCTTAGTACATACTATCAACAGGTTGAACTGCTGATCTTAATTAAAATTATCTCTAAGGCATGTGAACTGGCTG
```

Supporting Figure 2. *A,* Construct design highlighting the location of gRNAs driven by U6 promoter, and dCas9-V5-APEX2 fusion driven by CMV promoter (PCMV) as well as SV40 promoter (PSV40)-controlled GFP-T2A-Puro (GTP). *piggyBac* inverted terminal repeats (ITR) sequences are boxed in blue. *B*, complete sequence of Zeo-pT-U6-ChoREgRNA-dCas9-APEX2-GTP.

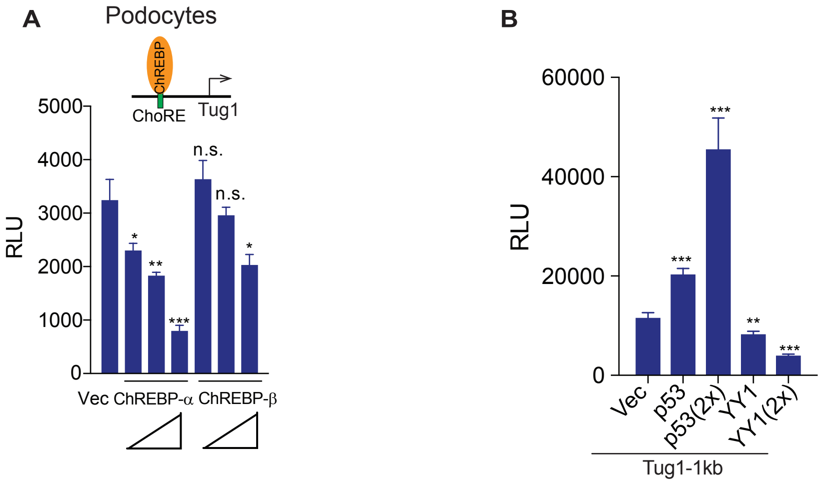

Supporting Figure 3. Effect of ChREBP-β, p53 and YY1 on Tug1 transcription. ***A,*** Luciferase activities in podocytes co-transfected with Tug1-1kb promoter reporter construct (Tug1-1kb) and increasing amounts of ChREBP-α or ChREBP-β cDNA expression constructs or empty pcDNA3 vector (Vec) under normal glucose conditions (n=3). Luciferase readings are presented as mean±s.e.m. *n.s.*, not significant, *, p<0.05, **, p<0.01, ***, p<0.001 (compared to vector group), ***B,*** Cultured mouse podocytes were transfected with Tug1-1kb promoter luciferase construct (Tug1-1kb), together with plasmids for overexpression of indicated transcription factors, or empty pcDNA3 vector (Vec) under normal glucose conditions. Luciferase assys were performed as in *A* (n=3).
